# STINGing away the pain: the role of interferon-stimulated genes

**DOI:** 10.1172/JCI180497

**Published:** 2024-05-01

**Authors:** Erick J. Rodriguez-Palma, Heather N. Allen, Rajesh Khanna

**Affiliations:** Department of Pharmacology and Therapeutics, College of Medicine, University of Florida, Gainesville, Florida, USA.

## Abstract

Pain and inflammation are biologically intertwined responses that warn the body of potential danger. In this issue of the *JCI*, Defaye, Bradaia, and colleagues identified a functional link between inflammation and pain, demonstrating that inflammation-induced activation of stimulator of IFN genes (STING) in dorsal root ganglia nociceptors reduced pain-like behaviors in a rodent model of inflammatory pain. Utilizing mice with a gain-of-function STING mutation, Defaye, Bradaia, and colleagues identified type I IFN regulation of voltage-gated potassium channels as the mechanism of this pain relief. Further investigation into mechanisms by which proinflammatory pathways can reduce pain may reveal druggable targets and insights into new approaches for treating persistent pain.

## Role of the STING/IFN-I pathway in pain signaling

Over the past decade, it has been demonstrated that the immune and sensory nervous systems closely collaborate to safeguard the host from an injury. This defense strategy is achieved by promptly detecting and recognizing potential danger that threatens the integrity of the individual (1). Key players in this mechanism involves the pattern recognition receptors (PRRs), specialized proteins responsible for detecting exogenous pathogens. The PRR family comprises Toll-like receptors, including retinoic acid–inducible gene I-like receptors, nucleotide oligomerization domain-like receptors, C-type lectin receptors, RIG-I–like receptors, and cytosolic DNA sensors (2). In response to infection or tissue injury, PRRs on immune cells are activated to initiate an inflammatory response, which in turn activates sensory neurons. However, sensory neurons are also able to sense danger signals after injury or during infection through the expression of PPRs (2, 3). In this context, stimulator of interferon genes (STING), a cytosolic DNA sensor that recognizes self-DNA, viral DNA, and cyclic dinucleotides produced by bacteria, has emerged as a regulator of pain signaling (4). Pharmacological activation of STING exerts antinociceptive effects in neuropathic mice (5–7), while deletion of the gene that encodes STING results in the development of mechanical allodynia (5). Furthermore, STING activation results in the production of type I IFNs (IFN-I) in immune cells and sensory neurons following infections or tissue injury (5, 8). Certainly, the production of proinflammatory mediators induces pain, but there is evidence indicating that inflammation can contribute to pain resolution by inducing antiinflammatory and proresolution mediators (9). In this sense, the STING/IFN-I signaling pathway has been widely debated; while some studies have demonstrated that STING agonist or IFN-I produce antinociceptive actions in the central nervous system (5, 10), the peripheral injection of STING agonist or IFN-I triggers nociceptive behaviors (11–13), suggesting that STING agonism and IFN-I can be proinflammatory and antiinflammatory, depending on context. Although previous studies have supported the notion that STING and IFN-I exhibit a relevant role in the pain signaling, the mechanisms by which the STING/IFN-I pathway modulates and reprograms nociceptors to promote the resolution of pain are yet to be elucidated.

## IFN-regulated genes in the resolution of inflammatory pain

**Figure 1:**
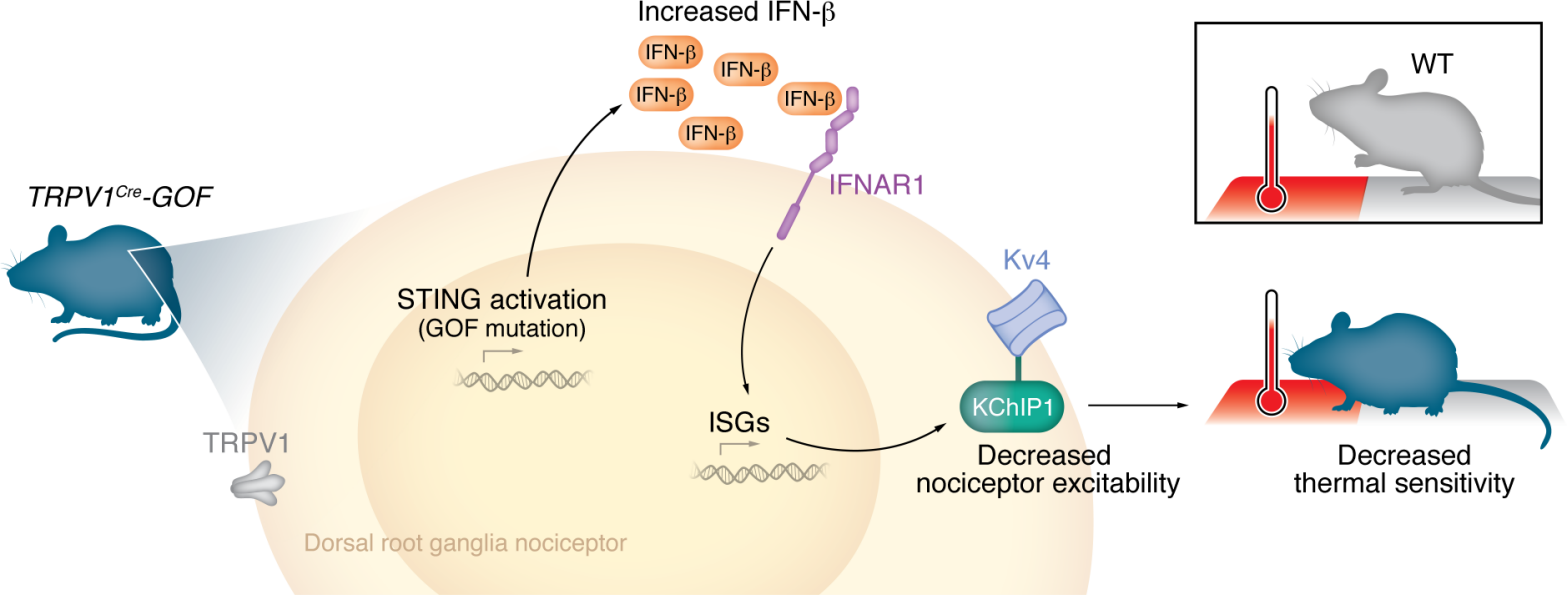
The STING/IFN-1 pathway regulates pain resolution. Mice expressing a STING gain-of-function (GOF) mutation in TRPV1 neurons display increased IFN-β levels. IFN signaling increases expression of ISGs, including *Kchip1*. Decreased excitability of nociceptors via regulation of KChIP-Kv4 interaction ultimately decreases thermal sensitivity.

In this issue of the *JCI*, Defaye, Bradaia, and colleagues shed light on how the STING/IFN-I pathway regulates ion channels and ion channel–associated proteins, contributing to the resolution of inflammatory pain (14). To understand how nociceptors respond to inflammatory milieu, Defaye, Bradaia, and colleagues utilized the well-established complete Freund’s adjuvant (CFA) model of inflammatory pain, which triggers local inflammation and produces pain-like behaviors. Transcriptional analysis in sensitized sensory neurons from CFA-treated mice revealed an increase in STING expression in Nav1.8^+^ and TRPV1^+^ neurons, indicating its upregulation in response to inflammation. Previous studies have demonstrated that STING activation in immune cells and/or sensory neurons leads to the release of IFN-I (5, 8). Notably, Defaye, Bradaia, and colleagues (14) demonstrated that deleting TRPV1^+^ neurons resulted in a 50% reduction in IFN-β, but not IFN-α, production in response to a STING agonist, suggesting that a substantial amount of IFN-I originates from TRPV1^+^ neurons. Additionally, intrathecal delivery of a neutralizing antibody against IFN-β delayed the resolution of CFA-induced thermal hyperalgesia, supporting the idea that IFN-β plays a role in pain resolution. To investigate the specific role of IFN-I in neurons, Defaye, Bradaia, and colleagues (14) expressed a STING gain-of-function (GOF) mutation in TRPV1^+^ neurons (referred to as *TRPV1^cre^-GOF*), resulting in constant production of IFN-β. This increase in IFN-β correlated with reduced thermal sensitivity, but not mechanical sensitivity, under inflammatory conditions. In this context, inhibition of IFNAR1 (via antibody block of IFN-α and IFN-β receptors) restored thermal hyperalgesia in CFA-treated *TRPV1^cre^-GOF* mice. Collectively, these findings suggest that the activation of neuronal STING alleviates inflammatory pain, partly through IFN-I/IFNAR1 signaling. It has been reported that IFN-stimulated genes (ISGs) enable host defense and facilitate recovery from inflammation (15). However, whether these genes played a relevant role in the resolution of pain during inflammation was unknown. Defaye, Bradaia, and colleagues (14) observed that constitutive activation of STING in TRPV1^+^ neurons led to a decrease in TRPV1, TRPA1, and TRPC3 expression, while promoting an increase in the A-type potassium (Kv) channel–regulating protein (KChIP1). Electrophysiological recordings assessing the functional impact of ISGs revealed that persistent STING activation and IFN-I production reduced the excitability of TRPV1 neurons by increasing Kv4-mediated A-type currents. These findings suggest that the STING/IFN-I pathway regulates ion channel expression, resulting in a reduction of sensory neuron excitability. Additionally, depletion of IFNAR1 or treatment with a specific Kv4 channel blocker in *TRPV1^cre^-GOF* neurons reduced neuronal excitability by decreasing Kv4-mediated A-type currents, suggesting that upregulation of Kv4 currents depends on IFN-I. To determine whether the Kv4-KChIP1 subunit complex underlies the STING/INF-I antinociceptive effect, the authors used a TAT-conjugated KChIP1 interfering peptide to disrupt functional Kv4-KChIP1 complexes. In vitro and in vivo assays revealed that the KChIP1 peptide promoted a reduction in the rheobase in *TRPV1^cre^-GOF* neurons, correlating with the development of thermal hyperalgesia in *TRPV1^cre^-GOF* mice after CFA injection. These findings indicate that the interaction between Kv4 and KChIP1 is essential for the antinociceptive effect induced by IFN-I. Overall, these findings highlight the importance of neuronal IFN-I as a crucial regulator of ion channels and channel-interacting proteins in sensory neurons during inflammatory pain (Figure 1).

## Future considerations and conclusions

The involvement of the STING/IFN-I signaling pathway in the context of inflammatory pain identified in Defaye et al. (14) is particularly relevant when considering that certain pathogens have developed mechanisms to directly activate sensory neurons, resulting in pain. IFN-I is induced by viral infections to protect the host against infection (16). A recent study demonstrated that IFN-I production is controlled by STING activation in sensory neurons and the STING/IFN-I/IFNAR axis plays a relevant role in pain signaling (5). It should be acknowledged that the role of IFN-I is still controversial, with reports suggesting both pronociceptive and antinociceptive actions of IFN-β and IFN-α (16). In this context, the work by Defaye, Bradaia, and colleagues elegantly demonstrates that upregulation of Kv4-mediated A-type currents in TRPV1^+^ neurons is a potential mechanism underlying the thermal hyperalgesia resolution during inflammatory pain (14). Importantly, this study raises several intriguing questions worthy of further investigation. What is the effect of STING activation in Nav1.8^+^ neurons? Does unchecked STING activation in nociceptors (i.e., cells expressing Nav1.8) exert an antinociceptive or pronociceptive effect? Answering these questions will help determine whether STING activation elicits different phenotypes in different neuronal subtypes. Considering that the increase in IFN-α, but not IFN-β, production was correlated with develop of mechanical allodynia, analyzing the effects of STING in Nav1.8 neurons is especially pertinent (13). Additionally, it is important to consider that inflammatory pain serves as a reminder of recent or ongoing injury, facilitating quick recovery. Therefore, further investigation is necessary to determine whether IFN-regulated genes, as consequence of STING activation, play a relevant role in the chronic inflammatory conditions.

## Version 1 05/01/2024

Electronic publication
